# Aiding the Diagnosis of Diabetic and Hypertensive Retinopathy Using Artificial Intelligence-Based Semantic Segmentation

**DOI:** 10.3390/jcm8091446

**Published:** 2019-09-11

**Authors:** Muhammad Arsalan, Muhammad Owais, Tahir Mahmood, Se Woon Cho, Kang Ryoung Park

**Affiliations:** Division of Electronics and Electrical Engineering, Dongguk University, 30 Pildong-ro 1-gil, Jung-gu, Seoul 04620, Korea; arsal@dongguk.edu (M.A.); malikowais266@gmail.com (M.O.); tahirmahmood.cs@gmail.com (T.M.); jsu319@naver.com (S.W.C.)

**Keywords:** diabetic retinopathy, retinal vessels, vessel segmentation, Vess-Net

## Abstract

Automatic segmentation of retinal images is an important task in computer-assisted medical image analysis for the diagnosis of diseases such as hypertension, diabetic and hypertensive retinopathy, and arteriosclerosis. Among the diseases, diabetic retinopathy, which is the leading cause of vision detachment, can be diagnosed early through the detection of retinal vessels. The manual detection of these retinal vessels is a time-consuming process that can be automated with the help of artificial intelligence with deep learning. The detection of vessels is difficult due to intensity variation and noise from non-ideal imaging. Although there are deep learning approaches for vessel segmentation, these methods require many trainable parameters, which increase the network complexity. To address these issues, this paper presents a dual-residual-stream-based vessel segmentation network (Vess-Net), which is not as deep as conventional semantic segmentation networks, but provides good segmentation with few trainable parameters and layers. The method takes advantage of artificial intelligence for semantic segmentation to aid the diagnosis of retinopathy. To evaluate the proposed Vess-Net method, experiments were conducted with three publicly available datasets for vessel segmentation: digital retinal images for vessel extraction (DRIVE), the Child Heart Health Study in England (CHASE-DB1), and structured analysis of retina (STARE). Experimental results show that Vess-Net achieved superior performance for all datasets with sensitivity (Se), specificity (Sp), area under the curve (AUC), and accuracy (Acc) of 80.22%, 98.1%, 98.2%, and 96.55% for DRVIE; 82.06%, 98.41%, 98.0%, and 97.26% for CHASE-DB1; and 85.26%, 97.91%, 98.83%, and 96.97% for STARE dataset.

## 1. Introduction

The segmentation of blood vessels from retinal images is a difficult and time-consuming task for medical specialists when diagnosing diseases such as hypertension, diabetic retinopathy, and arteriosclerosis [1]. Retinal vasculature is considered a unique entity to examine the structural and pathological changes related to ophthalmic diseases (glaucoma, diabetic retinopathy, hypertension, age-related macular degeneration etc.). The measurement and analysis of retinal vessels can be used as a biomarker for the diagnosis of cardio patients [2], similar to how homocysteine is used as a biomarker for diabetic retinopathy (DR) [3], which is the leading cause of vision loss [4]. Correct retinal vessel segmentation provides the opportunity for early diagnosis of diabetic retinopathy, which can later lead to blindness, and also helps to localize the position of optical discs and fovea [5]. Considering the importance of correct vessel segmentation, algorithms have been developed for image registration to find dissimilarities between two retinal images, which requires the use of a pre-segmentation method [6]. Finding the accurate tortuosity of retinal vessels is an ophthalmological diagnostic constraint, which depends on the vessel skeleton segmentation methods for curvature and thickness [7]. Diabetic retinopathy is instigated by high blood-sugar level, which causes leaking and swelling of retinal vessels. It can cause growth or creation of new blood vessels and can be detected by medical specialists using vascular analysis [8,9]. Hypertensive retinopathy is another retinal disease that is caused by high blood pressure in a hypertensive patient, which causes the shrinking of retinal blood vessels [10,11]. The manual segmentation of this valuable vascular structure is an intensive task, where accuracy and speed are important constraints [12]. Retinal blood vessels have a very complex structure with various tortuosity and shape. This and low-quality imaging make the segmentation difficult [13]. Researchers have proposed several approaches to automatically segment blood vessels from fundus images, but due to uneven pixel intensities of the vessels, this is challenging in terms of speed and accuracy. The success of artificial intelligence using deep learning has made medical analyses easier for medical professionals [14,15,16,17,18,19,20,21,22]. For robust vessel detection, this study proposes a residual mesh-based vessel segmentation network (Vess-Net) that can segment true vessels in fundus images, where residual dual streams maintain the feature re-use policy inside the network. The retinal images are of inferior quality and have low intensities. The continuous convolutional process for low-quality imaging causes the loss of important information. Our Vess-Net is a fully convolutional neural network (FCN) that avoids loss using a dual-stream information flow (internal and external). A detailed discussion on the Vess-Net structure and operation is presented in Section 4.

The rest of the paper is structured as follows: Section 2 provides brief information on related works. Section 3 describes the contribution of the paper. Section 4 explains the structure and methodology of the proposed method. Section 5 presents the experimental results. Section 6 provides the conclusion and future directions of the proposed work.

## 2. Related Works

Vessel segmentation can be divided into two main groups: techniques based on conventional handcrafted local features using typical image-processing schemes and techniques that use machine learning or deep-learning features.

### 2.1. Vessel Segmentation Based on Conventional Handcrafted Local Features

These methods use conventional image-processing schemes to identify vessels in fundus images. The usual schemes are color-based segmentation, adaptive thresholding, morphological schemes, and other local handcrafted feature-based methods that use image enhancement prior to the segmentation. Akram et al. used a 2D Gabor filter for retinal image enhancement and the multi-layer thresholding approach to detect blood vessels [23]. Fraz et al. used quantitative analysis of retinal vessel topology and size (QUARTZ), where vessel segmentation is carried out using a line detection scheme in combination with hysteresis morphological reconstruction based on a bi-threshold procedure [24]. Kar et al. used automatic blood vessel extraction using a matched filtering-based integrated system, which uses a curvelet transform and fuzzy c-means algorithm to separate vessels from the background [25]. Another recent example of an unsupervised approach was illustrated by Zhao et al., who used a framework with three steps. In the first step, a non-local total variation model adapted to the Retinex theory is used. In the second step, the image is divided into super-pixels to locate the object of interest. Finally, the segmentation task is performed using an infinite active contour model [26]. Pandey et al. used two separate approaches to segment thin and thick blood vessels. To segment thin blood vessels, local phase-preserving de-noising is used in combination with line detection, local normalization, and entropy thresholding. Thick vessels are segmented by maximum entropy thresholding [27]. Neto et al. proposed an unsupervised coarse-to-fine method for the blood vessel segmentation. Image enhancement schemes, such as Gaussian smoothing, morphological top-hat filtering, and contrast enhancement, are first used to increase the contrast and reduce the noise, and then the segmentation task is carried out via adaptive local thresholding [28]. Sundaram et al. proposed a hybrid segmentation approach that uses techniques such as morphology, multi-scale vessel enhancement, and image fusion i.e., area-based morphology and thresholding are used to highlight blood vessels [29]. Zhao et al. proposed an infinite active contour model to automatically segment retinal blood vessels, where hybrid region information of the image is used for small vasculature structure [30]. To detect vessels rapidly and accurately, Jiang et al. proposed a global thresholding-based morphological method, where capillaries are detected using centerline detection [31]. Rodrigues et al. performed vessel segmentation based on the wavelet transform and mathematical morphology, where tubular properties of blood vessels were used to detect retinal veins and arteries [32]. Sazak et al. proposed a retinal blood vessel image-enhancement method in order to increase the segmentation accuracy. They used the multi-scale bowler-hat transform based on mathematical morphology, where vessel-like structures are detected by thresholding after combining different structuring elements [33]. Chalakkal et al. proposed a retinal vessel segmentation method using the curvelet transform in combination with line operators to enhance the contrast between the background and blood vessels; they used multiple steps of conventional image processing such as adaptive histogram equalization, diffusion filtering, and color space transformations [34]. Wahid et al. used multiple levels to enhance retinal images for segmentation. In their technique, the enhanced image is subtracted from the input image iteratively, resultant images are fused to create one image, and this image is then enhanced using contrast-limited adaptive histogram equalization (CLAHE) and fuzzy histogram-based equalization (FHBE). Finally, thresholding is used to segment the enhanced image [35]. Ahamed et al. also applied CLAHE with the green channel of fundus images and used a multiscale line detection approach in combination with hysteresis thresholding; the results in this technique are refined by morphology [36]. 

### 2.2. Vessel Segmentation Using Machine Learning or Deep Learning (CNN)

Methods based on handcrafted local features have a limited performance. In addition, the performance is affected by the type of database. Therefore, machine learning or deep learning-based methods have been researched as an alternative. Zhang et al. used a supervised learning method for vessel segmentation. They used the anisotropic wavelet transform, where a 2D image is lifted to a 3D image that provides orientation and position information. Then, a random forest classifier is trained to segment retinal vessels from the background [37]. Tan et al. proposed a single neural network to segment optic discs, fovea, and blood vessels from retinal images. The algorithm passes the three channels of input from the point’s neighborhood to the seven-layer convolutional neural network (CNN) to classify the candidate class [38]. Zhu et al. proposed a supervised method based on extreme machine learning (EML), which utilizes a 39D vector with features such as morphology, divergence field, hessian features, phase congruency, and discriminative features. These features are then classified by EML, which extracts the vasculature from the background [39]. Wang et al. proposed a cascade classification method for retinal vessel segmentation. They iteratively trained a Mahalanobis distance classifier with a one-pass feed-forward process to classify the vessels and background [40]. Tuba et al. proposed support vector machine (SVM)-based classification using chromaticity and coefficients of the discrete cosine transform as features. The green channel from retinal images was used as the base of these features as it has maximum vessel information [41]. Savelli et al. presented a novel approach to segment vessels that corrected the illumination. Dehazing was used as a pre-processing technique to avoid haze and shadow noise, and classification was performed by a CNN that was trained on 800,000 patches with a dimension of 27 × 27 (the center pixel was considered the decision pixel) [42]. Girard et al. proposed a fast deep learning method to segment vessels using a U-Net-inspired CNN for semantic segmentation, where the encoder and decoder provide the down-sampling and up-sampling of the image, respectively [43]. Hu et al. proposed a method for retinal vessel segmentation based on a CNN and conditional random fields (CRFs). Basically, there are two phases in this method; in the first phase, a multiscale CNN architecture with improved cross-entropy loss function was applied to the image, then CRFs were applied to obtain the refined final result [44]. Fu et al. proposed DeepVessel, a program that uses deep learning in combination with CRFs. A multi-scale and multi-level CNN is used to learn rich hierarchical representations from images [45]. Soomro et al. proposed a deep-learning-based semantic segmentation network inspired by the famous SegNet architecture. In the first step, grayscale data were prepared by principle component analysis (PCA). In the second step, deep-learning-based semantic segmentation was applied to extract the vessels. Finally, post-processing was used to refine the segmentation [46]. Guo et al. proposed a multi-level and multi-scale approach, where short-cut connections were used for the semantic segmentation of vessels and semantic information was passed to forward layers to improve the performance [47]. Chudzik et al. proposed a two-stage method to segment retinal vessels. In the first step, the CNN is utilized to correlate the image with corresponding ground truth by random tree embedding. In the second stage, a codebook is created by passing the training patches through the CNN in the previous step; this codebook is used to arrange a generative nearest-neighbor search space for the feature vector [48]. Hajabdollahi et al. proposed a simple CNN-based segmentation with fully connected layers. These fully connected layers are quantized and the convolutional layers are pruned to increase the efficiency of the network [49]. Yan et al. proposed a three-stage CNN approach for vessel segmentation to improve the capability of vessel detection. The thick and thin vessels are treated by separate CNNs and the results are fused to produce a single image by a third CNN [50]. Soomro et al. proposed a semantic segmentation network based on modified U-Net, where the pooling layers are replaced by progressive convolution and deeper layers. In addition, dice loss is used as a loss function with stochastic gradient descent (SGD) [51]. Jin et al. proposed a deformable U-Net-based deep neural network. The deformable convolutions are integrated in the network and an up-sampling operator is used to increase the resolution of the image to extract more precise feature information [52]. Leopold et al. presented Pixel CNN with batch normalization (PixelBNN), which is based on U-Net and pixelCNN, where pre-processing is used to resize, reduce the dimension, and enhance the image [53]. Wang et al. used Dense U-Net as a semantic segmentation network for vessel segmentation, where random transformations are used for data augmentation in order to boost the effective patch-based training of the dense network [54]. Feng et al. proposed a cross-connected CNN (CcNet) for retinal vessel segmentation. The CcNet is trained on only the green channel of the fundus image; cross connections and fusion of multi-scale features improve the performance of the network [55]. However, in these previous works, deep networks were used, which included many trainable parameters that increased the network complexity. To address these issues, this paper presents a dual residual stream-based Vess-Net, which is not as deep as conventional semantic segmentation networks but provides good segmentation with few trainable parameters and layers. The method takes advantage of artificial intelligence in the process of semantic segmentation to aid the diagnosis of retinopathy.

Table 1 shows a comparison between existing methods and Vess-Net for vessel segmentation.

## 3. Contribution

This study paper on retinal vessel segmentation under challenging conditions to aid the process of diagnosis in retinopathy. Compared to previous works, our research presents the following novelties:

- Vess-Net performs semantic segmentation to detect retinal vessels without the requirement of conventional pre-processing.
- Vess-Net guarantees dual-stream spatial information flow inside and outside the encoder–decoder.
- Vess-Net’s internal residual skip path (IRSP) ensures feature re-use policy in order to compensate for spatial loss created by the continuous convolution process.
- Vess-Net’s outer residual skip path (ORSP) is designed to provide direct spatial edge information from the initial layer of encoder to the end of decoder. Moreover, the direct information flow pushes the Vess-Net to converge faster (in just 15 epochs with 3075 iterations).
- Vess-Net utilizes the benefits of both identity and non-identity mappings for outer and inner residual connections, respectively
- For fair comparison with other research results, the trained Vess-Net models and algorithms are made publicly available in [56].

## 4. Proposed Method

### 4.1. Overview of Proposed Architecture

**Figure 1:**
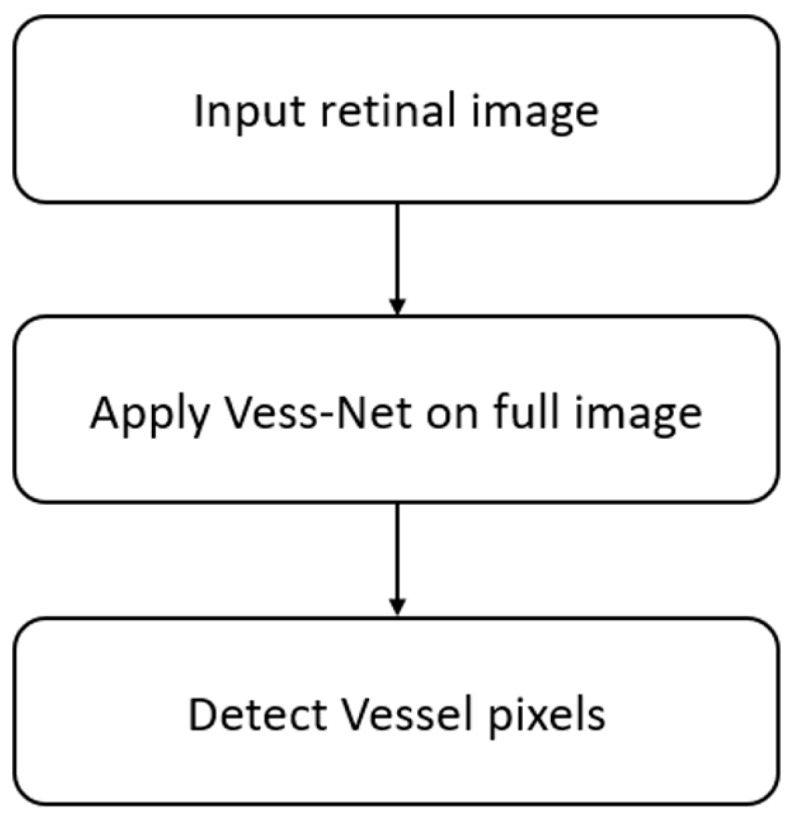
Flowchart of the proposed method.

Figure 1 shows an overview of the proposed method, which represents the overall semantic segmentation process in the detection of retinal blood vessels. Vess-Net provides accurate pixel-wise segmentation using a dual-stream spatial information flow provided by the residual mesh. The inner residual mesh compensates for the lost spatial information, whereas the outer residual mesh provides direct edge information from the initial layers to the end of the decoder. The Vess-Net takes the original image as the input without pre-processing and provides pixel-wise segmentation in an encoder–decoder manner.

### 4.2. Retinal Blood Vessel Segmentation Using Vess-Net

**Figure 2:**
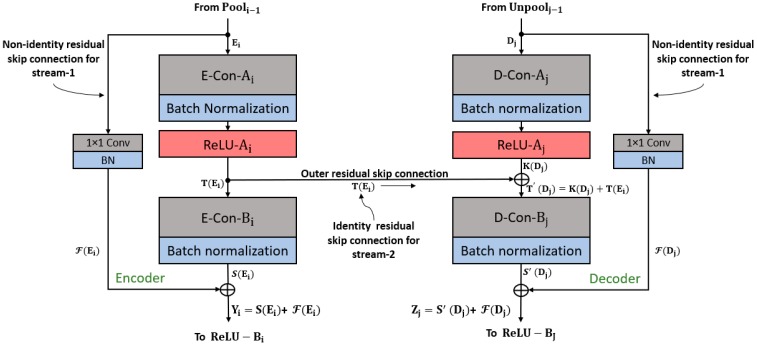
Vess-Net feature re-use schematic.

**Figure 3:**
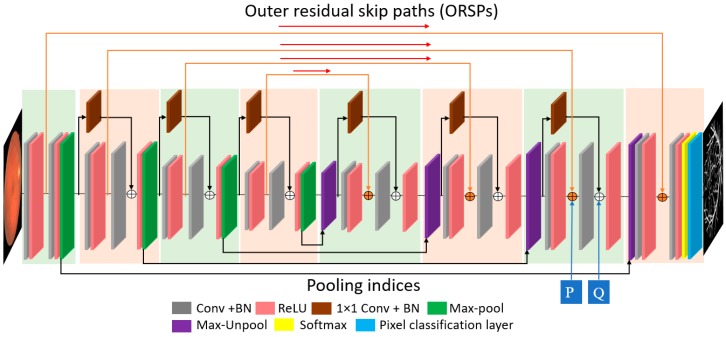
Proposed Vess-Net.

To extract retinal vessels from the input image, pixel-level classification is required; this is done via continuous convolution until the image is represented by its tiny features. The process of representing images with tiny features involves many convolutions, and in each convolution there is a loss of spatial information. Therefore, to obtain good classification accuracy, the image should not be excessively crushed during convolution [57]. Because deep analysis of retinal vasculature is required for disease diagnosis, the segmentation should be accurate even for tiny vessels. The usual network represents the image using a very small feature map (7 × 7), which is too small for minor information [57]. To detect retinal blood vessels in unconstrained scenarios, the network should maintain the lost spatial information throughout the network; it should be designed so that the feature map is sufficiently detailed to represent maximum features of the blood vessels. The Vess-Net has a minimum feature map size of 27 × 27 for a 447 × 447 input image, which is detailed enough to represent even tiny retinal vessel features. The image classification accuracy with residual networks (ResNet) [58] is higher due to the residual connectivity between the layers, which is beneficial for resolving the vanishing gradient problem [58]. ResNet improves the classification accuracy over visual geometry group networks (VGG nets), which do not use residual skip paths [58,59]. VGG net [59] is the base of SegNet, which also does not use residual skip connections [60]. Vess-Net is a 16-layer semantic segmentation network based on a fully residual encoder–decoder network (FRED-Net) [61]. FRED-Net utilizes the residual connectivity only inside the encoder and decoder, and there are no outer residual connections. The original ResNet [58] only uses the residual connectivity between adjacent layers and there is no decoder in the classification, whereas Vess-Net follows the two streams for the information flow with an encoder–decoder design. Stream 1 is a sequential layer–by-layer path in which adjacent layers of the encoder and decoder are connected by non-identity mapping (only), as shown in Figure 2 and Figure 3. Stream 2 is the external stream in which the encoder layers are directly connected with the corresponding decoder layers to provide high-frequency spatial edge information using identity mapping (only), as shown in Figure 2 and Figure 3. Figure 2 shows how the features are empowered by the two streams to detect tiny retinal blood vessels; the connectivity on the left and right is based on non-identity mapping to implement the feature re-use policy inside the encoder and decoder (Stream 1). In addition, the center connectivity represents Stream 2 which connects the encoder directly to the decoder with identity mapping. ResNet [58] uses both identity and non-identity mapping for classification before fully connected layers; Vess-Net uses a similar concept, but in a different way. It utilizes only non-identity mapping inside the encoder and decoder and only identity mapping for outer residual skip connections; in addition, the outer paths (identity) initiate and terminate inside the non-identity block, as shown in Figure 2 and Figure 3. In Figure 2, the connectivity of each convolution block is represented for both the encoder (left) and decoder (right); each convolution block takes the input, $E_{i}$/$D_{j}$, from the previous pooling/unpooling layer (${\mathrm{Pool}}_{i-1}$/${\mathrm{Unpool}}_{j-1}$ in Figure 2), and provides the empowered output, $Y_{i}/Z_{i}$, to the rectified linear unit $(\mathrm{ReLU}-B_{i}$/$\mathrm{ReLU}-B_{j})$ encoder/decoder. The first and second convolutional layers of encoder are represented by E-Con-$A_{i}$ and E-Con-$B_{i}$, respectively, with a batch normalization (BN) layer, whereas the first and second convolution layers in each decoder is represented by D-Con-$A_{j}$ and D-Con-$B_{j}$, respectively. $T(E_{i})$ and $K(D_{j})$ are the output features after the first convolution for the encoder and decoder, respectively, whereas $S(E_{i})$ and $S^{\prime }(D_{j})$ are the output features after the second convolution for the encoder and decoder, respectively. 

According Figure 3, the first and the last convolution blocks do not have an inner residual connection because these are the input and output convolutional blocks, respectively, as described in [61]. As stated above, every second ReLU in each convolutional block (except the first) in the encoder receives an empowered feature $Y_{i}$, which is the resultant feature after the element-wise addition of $F(E_{i})$ and $S(E_{i})$ by the non-identity residual skip path shown in Figure 2; this is given by the following equation:

(1) $$Y_{i}=S(E_{i})\;+\;F(E_{i})$$

The “+” sign indicates an element-wise addition and $Y_{i}\;$ is the enhanced feature available for the activation (ReLU-B_i_) after the element-wise addition of features from E-Con-B_i_ ($\mathrm{after}\;\mathrm{the}\;\sec\mathrm{ond}\;\mathrm{convolution},\;S(E_{i})$) and the feature from the 1 × 1 convolution in the residual skip path ($F(E_{i})$). For the decoder, the situation is completely different because of the outer residual path (non-identity mapping, shown in the middle of Figure 2); the element-wise addition of this feature inside the decoder-side convolutional block provides quality spatial-edge information. At the decoder side, each second convolution obtains the edge–information-enriched feature by identity mapping. This enriched feature, $T^{\prime }(D_{j})$ (for example after Point “P” in Figure 3) is available for each second convolution in the decoder block (D-Con-$B_{j}$) and is given by Equation (2):

(2) $$T^{\prime }(D_{j})=K(D_{j})\;+\;T(E_{i})$$

$T^{\prime }(D_{j})$ is the enriched feature with the spatial edge information. It is the element-wise addition of $K(D_{j})$ and $T(E_{i})$, which are, respectively, the output feature after the first activation of each decoder block (ReLU-$A_{j}$) and Stream 2 features that are directly imported after the first activation of each encoder block (ReLU-$A_{i}$) by identity mapping. $T^{\prime }(D_{j})$ is available for each second convolution (except the last) in the decoder represented by D-Con-$B_{j}$ in Figure 2. As Vess-Net also involves a dual-stream feature empowerment, the enriched feature $T^{\prime }(D_{j})$ is further element-wisely added to Stream 1 ($F(E_{i})$ by non-identity mapping for maximum benefit of the feature re-use policy. According to Figure 2, each second ReLU activation in the decoder (ReLU-B_j_) receives a dual-stream enhanced feature, $Z_{j}$, given by the equation:

(3) $$Z_{j}=S^{\prime }(D_{j})+\;F(D_{j})\;$$

Here, $Z_{j}$ is a dual-stream enhanced feature by both identity and non-identity mapping (for example after Point “Q” in Figure (3)). In addition, $S^{\prime }(D_{j})$ is the output feature from each second convolution (except the last) in the decoder (D-Con-$B_{j}$) and $F(D_{j})$ is the feature from the non-identity residual skip path.

Each outer residual skip path (ORSP-1 to ORSP-4) provides a $T(E_{i})$ feature to the corresponding decoder block. $Z_{j}$ is the empowered feature from the inner and outer streams that improves the network capabilities to segment minor features of retinal blood vessels.

#### 4.2.1. Vess-Net Encoder

As shown in Figure 3, the Vess-Net has a total of 16 convolution layers of 3 × 3 size; eight convolution layers are for the encoder and eight for the decoder. Insider the Vess-Net encoder, there are four convolutional blocks, each containing only two convolutional layers, as shown in Figure 3. The number of convolution layers is directly proportional to the depth of the network and number of trainable parameters. Each of the convolutional block in the encoder contains one pooling layer (Pool1–Pool4) in order to down-sample the feature. In addition, these pooling layers provide indices and image size information to the decoder to maintain the feature map at the decoder end. Each convolutional layer has a BN layer and ReLU layer for activation. The non-identity skip connections in the encoder start right after each pooling layer of current convolutional blocks and terminate immediately after the second convolution of next convolutional block in order to avoid the loss of spatial information during the convolution process. The identity skip connections are initiated after the first ReLU of each encoder convolutional block; these paths for identity mapping are directly provided to the corresponding decoder block to provide the spatial edge information. Due to the dual-stream feature empowerment, the Vess-Net encoder provides valuable features to the decoder for up-sampling. Table 2 represents the notable differences between the proposed Vess-Net method, ResNet [58], IrisDenseNet [62], and FRED-Net [61].

The Vess-Net structure is described in Table 3, which shows that there are 10 residual connections that connect the encoder and decoder, both externally and internally. The inner residual skip connections provide the lost spatial information after the convolution process, whereas the outer path helps converge the network faster by immediately providing spatial information.

#### 4.2.2. Vess-Net Decoder

As explained in Figure 3, the function of the proposed Vess-Net decoder is to produce an output similar to the input image. To mimic back image, the decoder layers exactly act as the mirror of encoder. That is, the decoder layers perform the reflect action to the encoder and provide the output image size same as input. The Vess-Net decoder has several paths coming from the encoder. The four paths from each pooling layer of the encoder provide the indices and size information to each unpooling layer of the decoder; this helps maintain the feature map of each stage of the decoder according to its corresponding stage in the encoder. There are four outer residual skip paths (ORSP-1 to ORSP-4) from each first convolutional layer of encoder blocks; each ORSP terminates before the second ReLU activation in the decoder. The ORSP provide important spatial edge information to the corresponding decoder layer. In each decoder block, there are two additional layers for element-wise addition: for the IRSP via non-identity mapping (Stream 1) and for the ORSP via identity mapping (Stream 2). Both streams through the IRSP and ORSP are combined in the decoder to ensure reliable features for accurate semantic segmentation of retinal blood vessels in difficult scenarios. The output of the network is in the form of a two-channel mask. As we have two classes (vessel and non-vessel), the number of filters for the last convolutional layer in the decoder is set to 2, which produces two separate masks for vessel and non-vessel classes. The pixel classification layer and the softmax function at the end of decoder assign one predicted class to each pixel in the feature map. Table 4 represents the layer patterns of the proposed Vess-Net decoder.

## 5. Experimental Results

### 5.1. Experimental Data and Environment

**Figure 4:**
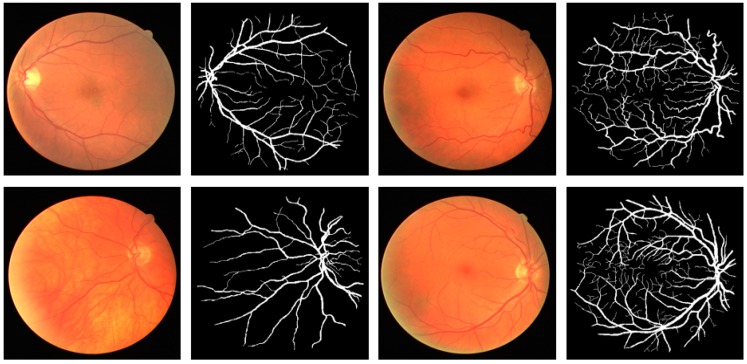
Sample fundus images and ground-truths for DRIVE dataset.

This research focused on retinal vessel segmentation. Therefore, the performance of the proposed Vess-Net method was tested over a publicly available dataset of digital retinal images for vessel extraction (DRIVE) [63]. The dataset consists of 40 RGB fundus camera images that are already divided equally for training and testing datasets (20 images for training and 20 images for testing). To test the algorithms and methods, the ground truth images of manually segmented retinal vessel are publicly available with the dataset and are provided in [63]. Figure 4 shows examples of images and the corresponding ground truth images for the DRIVE dataset.

In our experiment, we used the training and testing image as given in the dataset. To reduce the graphic processing unit (GPU) memory usage, the images and labels are resized to 447 × 447 pixels. Vess-Net is a semantic segmentation neural network that takes both image and annotation at the same time for the training. Moreover, for better training, we artificially increased the amount of data using image data augmentation, as described in Section 5.2.

The Vess-Net is trained and tested on an Intel^®^ Core™ i7-3770K CPU @ 3.50 GHz (4 cores), with 28 GB of system RAM, NVIDIA GeForce GTX Titan X GPU with 3072 Cuda cores, and a graphic memory of 12 GB (NVIDIA, Santa Clara, CA, USA) [64]. In our experiments, the proposed model is designed and trained from scratch on our experimental dataset using MATLAB R2019a [65]. Hence, there is no fine-tuning of any pre-trained model such as ResNet, GoogleNet, Inception, or DenseNet etc.

### 5.2. Data Augmentation

**Figure 5:**
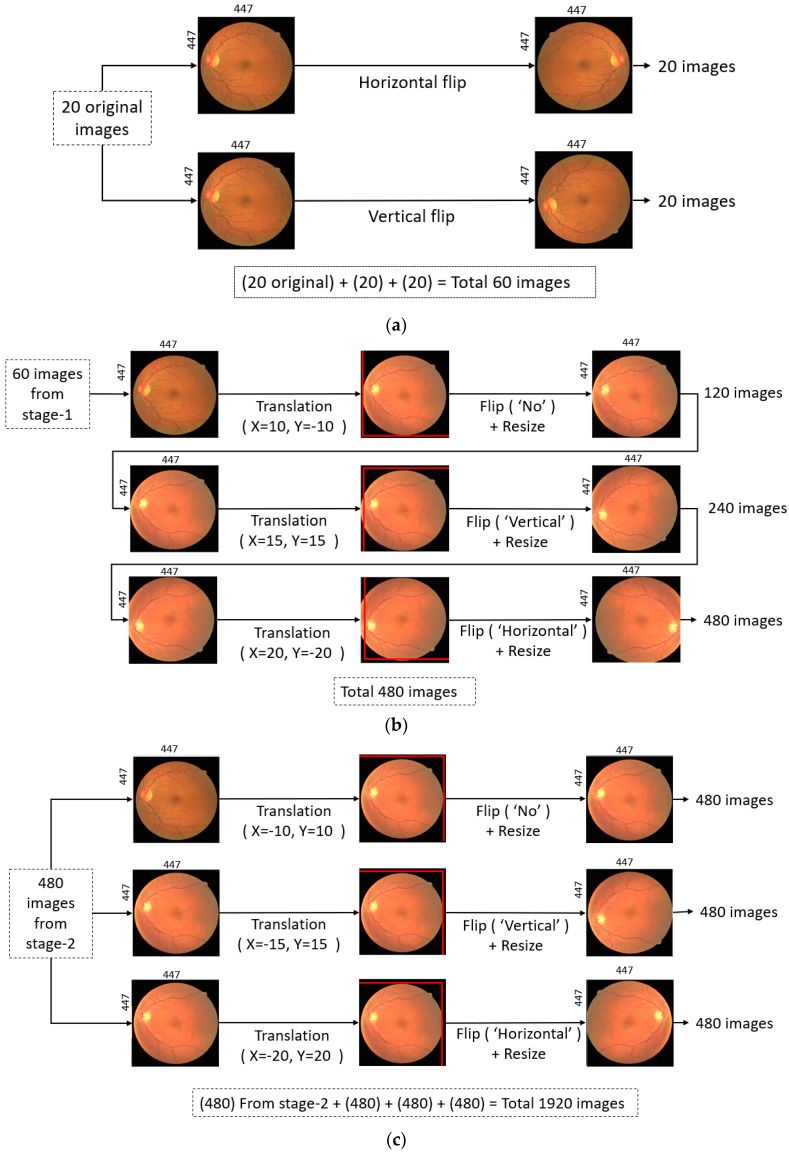
Data augmentation method: (**a**) Stage 1 augmentation by flipping; (**b**) Stage 2 augmentation by translation, flip, and resize (recursively); and (**c**) Stage 3 augmentation by translation, flip, and resize (non-recursively).

To sufficiently train Vess-Net, the training dataset should be large. It is very difficult to train a deep neural network with only 20 training images and obtain reliable segmentation. To train the Vess-Net properly and provide distinctive training examples, data from the 20 images with corresponding ground truths were augmented by artificially creating additional images. Various image transformations such as horizontal flipping, vertical flipping, and crop-resize with nearest neighbor interpolation were used, as shown in Figure 5. 

These transformations were performed in three stages. In Stage 1, 20 images were generated by a horizontal flip and 20 images were generated by a vertical flip, resulting in 60 images, as shown in Figure 5a. In Stage 2, the 60 images from Stage 1 were translated in the X-Y directions, flipped, and resized with three different combinations: (X = 10, Y = −10, “no-flip”, Resized), (X = 15, Y = 15, “Vertical flip”, Resized), and (X = 20, Y = −20, “Horizontal flip”, Resized). This generated 480 images, as shown in Figure 5b. In Stage 3, the 480 images from the previous stage were separately transformed using X-Y translation, flip, and resize with three different combinations: (X = −10, Y = 10, “no-flip”, Resized), (X = −15, Y = 15, “Vertical flip”, Resized), and (X = −20, Y = 20, “Horizontal flip”, Resized). Each step generated 480 additional images, resulting in a total of 1440 images for Stage 3. Hence, 480 images from the second stage (shown by Figure 5b) and 1440 images from third stage resulted in a total of 1920 for training, as shown in Figure 5c.

### 5.3. Vess-Net Training

**Figure 6:**
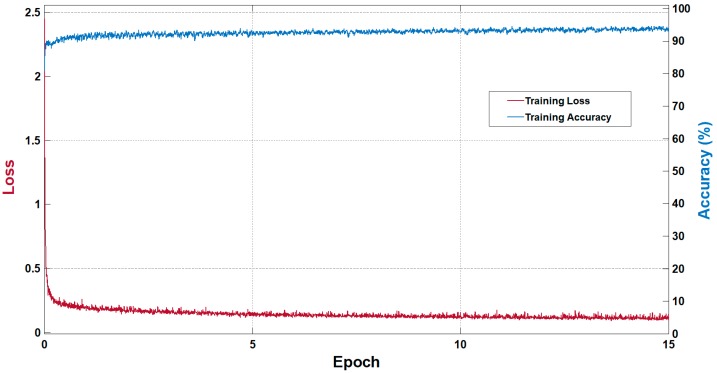
Training accuracy and loss curves for Vess-Net.

Vess-Net is a fully convolutional network with dual-stream information flow that allows it to converge faster. This type of connectivity allows the network to train without pre-processing of data. As Vess-Net is our own designed network, the training was performed from scratch without any weight initialization or fine-tuning. Adam was chosen as the optimizer as it is a more sophisticated version of the stochastic gradient descent (SGD) (SGD is based on a first-order gradient-based method). Adam is computationally more efficient compared to conventional SGD because of its efficacy for diagonal scaling of the gradients [66]. In this study, the Adam optimizer had an initial learning rate of 0.0005, which was maintained during the training with a mini-batch-size of seven images. The gradient threshold method using global L2 normalization with an epsilon of 0.000001 was adopted. As Vess-Net empowers features with dual stream, it was trained for only 15 epochs, with shuffling of images in each epoch to maintain the variety of learning. Cross-entropy loss was used with median-frequency class balancing to eliminate the effect of class imbalance, as described in [62]. Figure 6 shows the training accuracy and loss curves for Vess-Net. The x-axis represents the number of epochs, the left y-axis represents the training loss, and right y-axis represents the training accuracy. The represented accuracy and loss were based on the mini-batch, which shows the training accuracy and training loss per epoch, respectively. With training for 15 epochs and an initial learning rate of 0.0005, Vess-Net showed a training accuracy of 96% and training loss of approximately 0.06. Training for more epochs did not result in any further increase in accuracy or reduction in loss. As stated in Section 3, the Vess-Net trained models will be made publicly available for fair comparison with other studies via [56].

### 5.4. Testing of Proposed Method

#### 5.4.1. Vess-Net Testing for Retinal Vessel Segmentation

Vess-Net is trained without pre-processing, and the input image is directly provided for the testing phase without prior pre-processing. The testing image takes both streams (inner and outer) to empower features using six internal skip paths with non-identity mapping (IRSP-1 to IRSP-6) and four outer skip paths with identity mapping (ORSP-1 to ORSP-4). The output from Vess-Net provides two masks: for vessel and non-vessel classes. The last 3 × 3 convolution is with the two filters representing both classes. To evaluate and compare our proposed Vess-Net on the DRIVE dataset with other methods, we adopted sensitivity (Se), specificity (Sp), accuracy (Acc), and area under the curve (AUC) as evaluation metrics. The formulas for Se, Sp, and Acc are given by Equations (4)–(6):

(4) $$\mathrm{Se}=\;\frac{tp}{tp+fn}$$

(5) $$\mathrm{Sp}=\;\frac{tn}{tn+fp}$$

(6) $$\mathrm{Acc}=\frac{tp+tn}{tp+fp+fn+tn}$$

where *tp*, *fn*, *tn*, and *fp* are the numbers of true positives, false negatives, true negatives, and false positives, respectively. Here, *tp* is a pixel that is listed as a vessel pixel in the ground truth and predicted as a vessel pixel by our network, whereas *fn* is a pixel that is listed as a vessel pixel in ground truth but predicted by the network as a non-vessel pixel. *tn* is a pixel that is listed as a non-vessel pixel and correctly predicted as a non-vessel by the network, whereas *fp* is a non-vessel pixel in the ground truth and is predicted as a vessel pixel by our network. 

#### 5.4.2. Vessel Segmentation Results by Vess-Net

**Figure 7:**
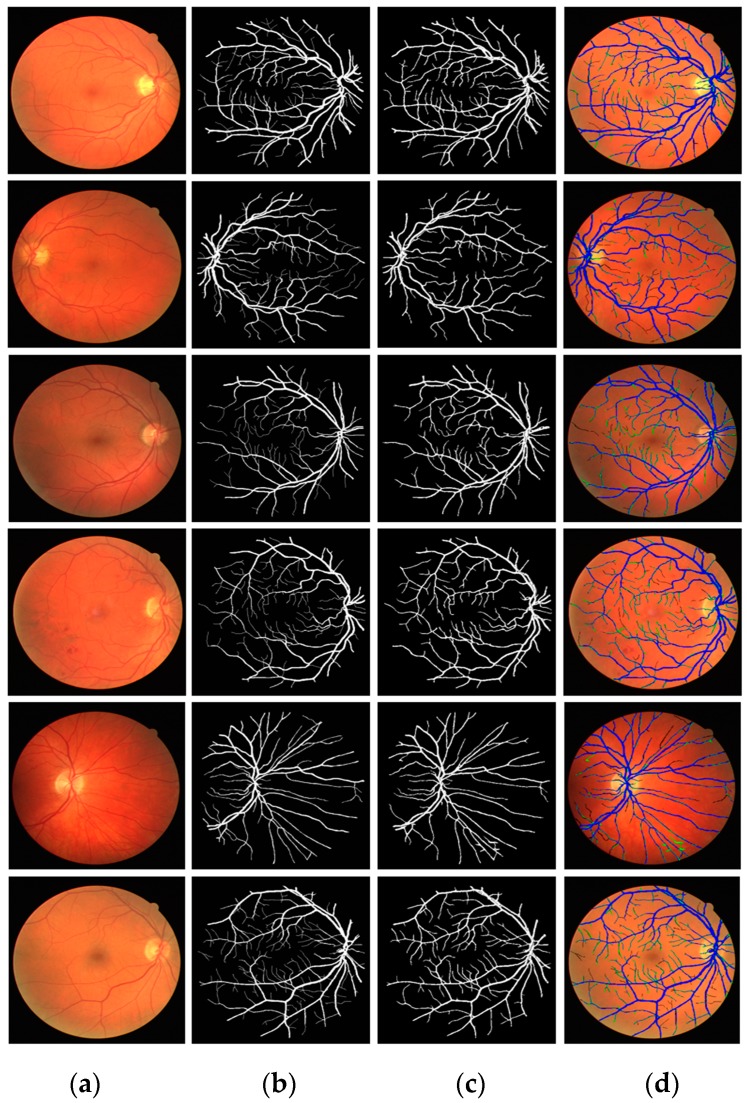
Examples of vessel segmentation by Vess-Net for DRIVE dataset: (**a**) original image; (**b**) ground-truth mask; (**c**) predicted mask by Vess-Net; (**d**) segmented image by Vess-Net (*tp* is presented in blue, *fp* in green, and *fn* in black).

Figure 7 presents the visual results of vessel segmentation by Vess-Net with the DRIVE dataset. Figure 7 includes cases of *fp* (shown in green), *fn* (shown in red), and *tp* (shown in blue). As shown in the figure, there was no significant error or no-segmentation case for the test images. 

#### 5.4.3. Comparison of Vess-Net with Previous Methods

This section provides comparisons between Vess-Net and other methods based on the evaluation metrics highlighted in Section 5.4.1. Table 5 presents the comparisons of the results obtained by local feature-based methods and learned feature-based methods with those obtained by Vess-Net for the DRIVE dataset. The results confirm the higher performance of Vess-Net for retinal vessel segmentation compared to existing methods, based on the values of AUC and Acc.

#### 5.4.4. Vessel Segmentation with Other Open Datasets Using Vess-Net

**Figure 8:**
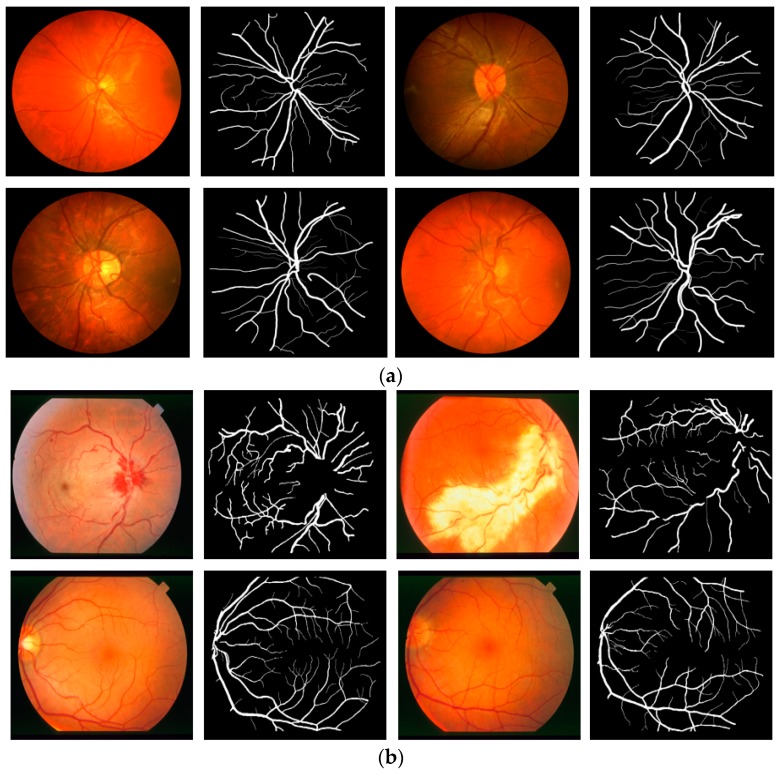
Examples of fundus images from (**a**) CHASE-DB1 and (**b**) STARE datasets with corresponding ground truths.

To evaluate the performance of Vess-Net in different situations, this study included experiments with two more open datasets: Child Heart Health Study in England (CHASE-DB1) [67] and structured analysis of retina (STARE) [68] for retinal vessel segmentation. CHASE-DB1 consists of l 28 images of 14 schoolchildren captured using a Nidek NM-200-D fundus camera with a 30° field of view. The STARE dataset consists of 20 images captured using a TopCon TRV-50 fundus camera with a 35° field of view. Examples of images for the CHASE-DB1 and STARE datasets are shown in Figure 8a,b, respectively. The manual segmentation mask for both CHASE-DB1 and STARE (first observer) were used as ground truths. In the experiment with CHASE-DB1, half of the images (14 images) were used for training with data augmentation (described in Section 5.2), while the other half (14 images) were used for testing with a two-fold cross validation. The overall performance was computed by averaging the two experimental results. For the STARE dataset, the experiment was repeated 20 times by selecting one image for testing and the other 19 images for training (leave-one-out validation) with data augmentation (described in Section 5.2). The overall performance was computed by averaging the 20 experimental results.

**Figure 9:**
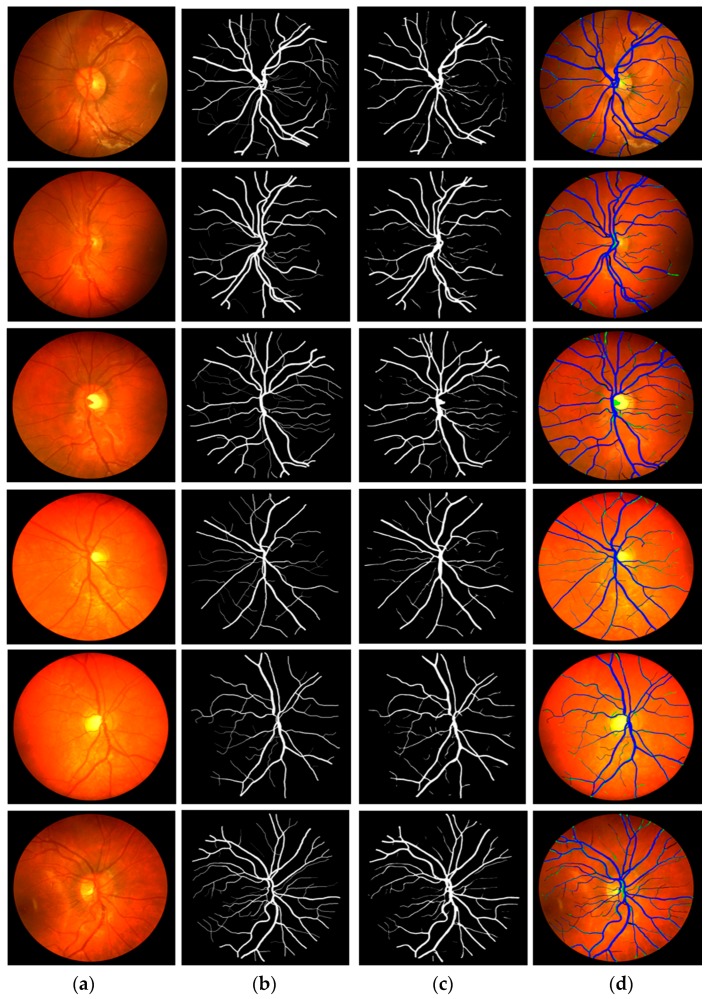
Examples of vessel segmentation by Vess-Net for CHASE-DB1: (**a**) original image; (**b**) ground-truth mask; (**c**) predicted mask by Vess-Net; (**d**) segmented image by Vess-Net (*tp* is presented in blue, *fp* in green, and *fn* in black).

**Figure 10:**
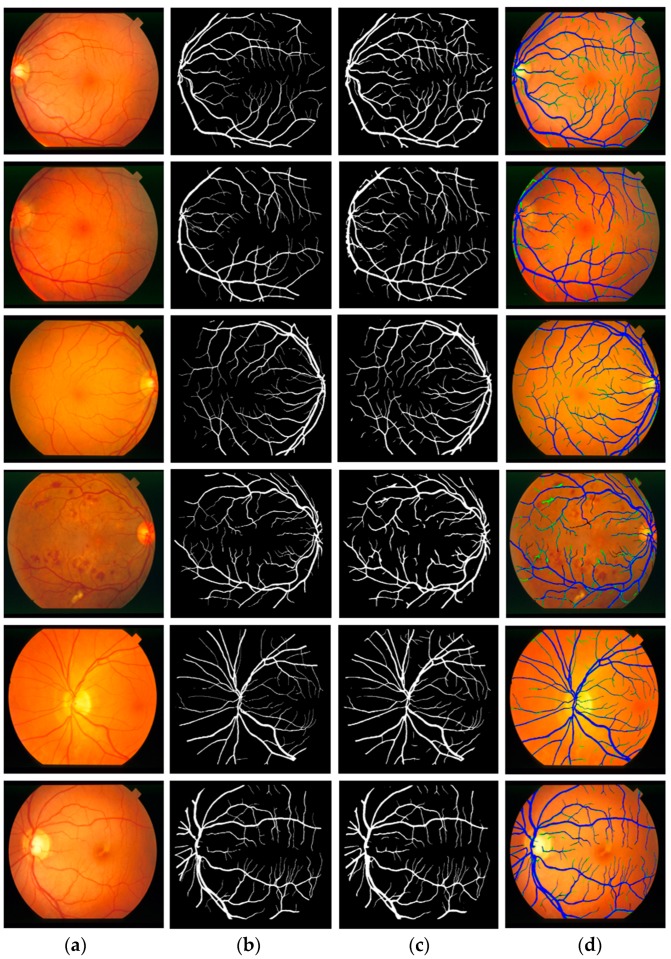
Examples of vessel segmentation by Vess-Net for STARE dataset: (**a**) original image; (**b**) ground-truth mask; (**c**) predicted mask by Vess-Net; (**d**) segmented image by Vess-Net (*tp* is presented in blue, *fp* in green, and *fn* in black).

Figure 9 and Figure 10 show the visual results of vessel segmentation and detection by Vess-Net with the CHASE-DB1 and STARE datasets. These figures also show *fp*, *fn*, and *tp*. There was no significant error or no-segmentation case for the test images. 

Table 6 and Table 7 present the comparison between local feature-based methods and learned feature-based methods and the proposed Vess-Net for CHASE-DB1 and STARE datasets, respectively. The results confirm the higher performance of Vess-Net for retinal vessel segmentation compared to existing methods.

The reason we performed the training and testing for the three separate databases is for the fair comparisons with the previous studies (based on same experimental protocol) as shown in Table 5, Table 6 and Table 7. As shown in these tables, our method outperformed the state-of-the-art methods with the three separate databases. To test the portability of our network, additional experiments were performed, in which the model was trained with the images of DRIVE [63] and CHASE-DB1 [67] datasets and tested with all the images of STARE dataset [68] independently. Table 8 shows the accuracies of Vess- Net. By comparing the accuracies in the case of three separate databases, as shown in Table 5, Table 6 and Table 7, with those of Table 8, the degradation of accuracies are very small, which confirms the portability of our network.

## 6. Detection of Diabetic or Hypertensive Retinopathy

**Figure 11:**
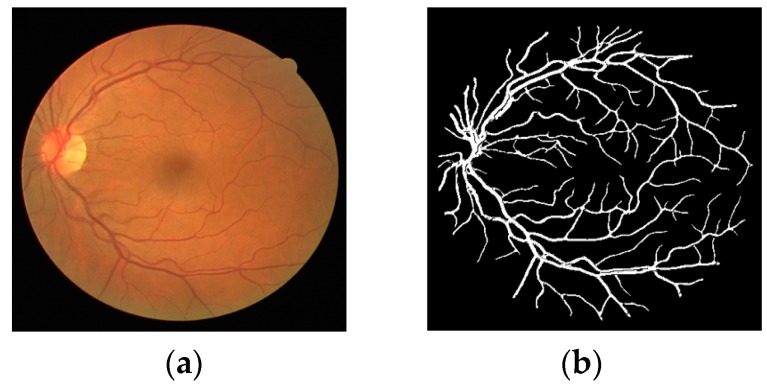
Sample image of vessel segmentation for pixel count: (**a**) original image; (**b**) predicted mask by Vess-Net.

As described in the Section 1, diabetic and hypertensive retinopathy can be detected with the help of accurate vessel segmentation, which is an intensive task for the medical specialist to do it manually [1]. Two retinal images of consecutive visits can be compared with the help of accurate segmentation algorithm. The proposed method provides accurate binary segmentation mask with pixel values of “0” and “1”. The number of pixels which are labeled as vessel pixel (marked as “1”) can be counted. If the number of vessel pixels is more than that of the previously registered image, it represents the swelling or creation of new blood vessels (which shows the presence of diabetic retinopathy [8,9]). If the number of vessel pixels is less than that of the previously registered image, it represents the shrinkage of blood vessels (which shows the presence of hypertensive retinopathy [10,11]). Figure 11 shows an example binary segmentation mask detected by the proposed method. In this case, the number of vessel pixels is 19,551 out of total 199,809 pixels in the image of 447 × 447 pixels. This pixel count can be used as biomarker to detect both diabetic and hypertensive retinopathy with appropriate threshold. However, the accuracy of this detection is totally dependent on the correct segmentation of vessels. The Se, Sp, AUC and Acc are the evaluation metrics to judge the correctness of the vessel segmentation which can produce the correct pixel count for the detection of diabetic or hypertensive retinopathy. The small vessels caused by the creation of new blood vessels are equally important to be segmented as these can be caused by the diabetic retinopathy, thus the segmentation algorithm should have sufficiently good performance to detect even small vessels. 

As shown in Figure 11, the ratio of non-vessel (negative data) vs. vessel pixels (positive data) is 9.2:1 (180,258 vs. 19,551). As shown in Equations (4) and (5), Se is calculated as the number of correctly detected vessel pixels (*tp*) over the number of whole vessel pixels (positive data), which shows the detection accuracy of vessel pixels, while Sp is calculated as the number of correctly detected non-vessel pixels (*tn*) over the number of whole non-vessel pixels (negative data), which shows the detection accuracy of non-vessel pixels. As shown in Table 5, Table 6, Table 7 and Table 8, Sp is higher than Se. However, in the experiments presented in Table 5, Table 6 and Table 7, we compared Sp, Se, and Acc (which shows the detection accuracy of both vessel and non-vessel pixels as shown in Equation (6)), which have been widely used as evaluation metric in previous studies, by our method with those by existing methods. In addition, as shown in these tables, our method outperformed the state-of-the-art methods.

As shown in Figure 7, Figure 9 and Figure 10, errors (*fn*) still exist for the small vessels by our method, but these errors can be compensated by the additional help of medical expert. For example, our system can be used as diagnostic method of the first step, and only the suspicious images can be checked again by medical expert as the second step. By using this scheme of two-step diagnosis, the correct prediction of diabetic and hypertensive retinopathy can be enhanced while lessening the diagnostic burden of medical expert.

## 7. Discussion

**Figure 12:**
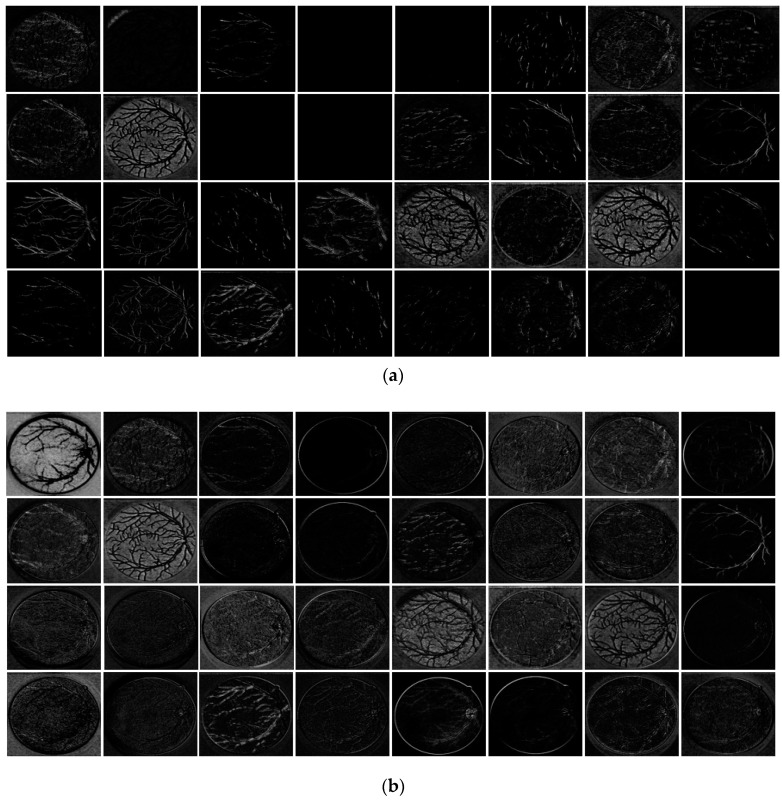

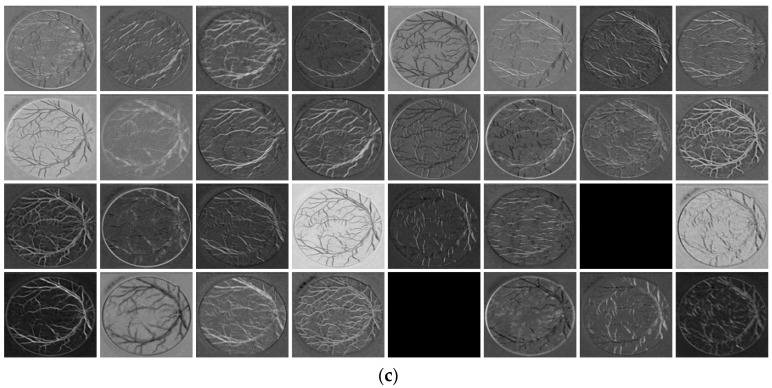
Dual-stream feature empowerment by Vess-Net: (**a**) features before point “P” (before Stream 2); (**b**) features after point “P” (after Stream 2); and (**c**) features after point “Q” (after both Streams 1 and 2).

In this study, a new technique is introduced to take advantage of the feature re-use policy. To compensate for the lost spatial information during the continuous convolution process and strengthen the feature traveling through the network, two residual streams are used. Stream 1 uses a series-type flow to empower the features by importing the information from the previous layer using non-identity skip paths, whereas Stream 2 is a direct stream from the encoder to decoder so that the edge information on each level has dedicated identity residual paths. To explain the effect of two streams, Figure 12 presents the feature maps from the decoder at three points. Points P and Q are from DCB-2, as shown in Table 4 and Figure 3. The feature maps are extracted before point P i.e., before Stream 2 (shown in Figure 12a), after point P i.e., empowered features with Stream 2 (shown in Figure 12b), and after point Q i.e., combined features of Streams 1 and 2 (shown in Figure 12c). Figure 12b,c clearly show that both streams significantly enhance the features for reliable segmentation of retinal vessels. Note that in Figure 12, the total number of DCB-2 channels is 128 but only the first 32 channels are shown for convenience. The important observations from our network are as follows:

- Vess-Net is empowered by IRSPs and ORSPs, which enables the network to provide high accuracy with few convolution layers (only 16).
- With provision of direct spatial edge information, the network is pushed to converge rapidly, i.e., in only 15 epochs (3075 iterations).
- Vess-Net is designed in a way that it maintains the minimal feature map size at 27 × 27 (as shown in Table 3), which is sufficient to represent tiny vessels that are created due to diabetic retinopathy.

## 8. Conclusions

This study proposed a dual-stream feature empowerment network (Vess-Net) for retinal vessel segmentation in non-ideal scenarios. The fundus images have very low pixel intensities for retinal vessels, which make them similar to the background and results in difficult segmentation. The proposed Vess-Net method has a two-way information flow that the differentiates between vessel and non-vessel classes even in the presence of continuous convolutions, which tend to lose spatial information in each stage. In the absence of these residual skip paths, tiny vessel information would be lost as the gradient vanishes and those tiny vessels are important for the diagnosis of diabetic retinopathy. To preserve tiny vessels, the incorporation of features from preceding layers results in a significantly enhanced segmentation process. Moreover, with this design, the network is powerful and can segment minor information with few layers. The direct connection from the encoder to decoder to provide edge information makes the network converge rapidly and substantially reduces the number of trainable parameters with fine segmentation of the vessels, which is important to compute vessel pixel count to detect the diabetic or hypertensive retinopathy. One of the most important characteristics of the proposed method is avoiding pre-processing overheads, so that original images can be provided to the network without conventional pre-processing for training and testing.

Vess-Net is creatively supported by inner and outer residual skip paths. Our future goal is to create a similar network with different mapping options that can provide a sufficiently good segmentation performance with fewer trainable parameters. In addition, this network can be used for semantic segmentation in various domains.

## Figures and Tables

**Table 1:** Comparison between Vess-Net and previous methods for vessel segmentation.

Type	Methods	Strength	Weakness
**Using handcrafted local features**	Vessel segmentation using thresholding [23,24,28,29,31,33,34,35,36]	Simple method to approximate vessel pixels	False points detected when vessel pixel values are closer to background
	Fuzzy-based segmentation [25]	Performs well with uniform pixel values	Intensive pre-processing is required to intensify blood vessels’ response
	Active contours [26,30]	Better approximation for detection of real boundaries	Iterative and time-consuming processes are required
	Vessel tubular properties-based method [32]	Good estimation of vessel-like structures	Limited by pixel discontinuities
	Line detection-based method [27]	Removing background helps reduce false skin-like pixels	
**Using features based on machine learning or deep learning**	Random forest classifier-based method [37]	Lighter method to classify pixels	Various transformations needed before classification to form features
	Patch-based CNN [38,42]	Better classification	Training and testing require long processing time
	SVM-based method [41]	Lower training time	Use of pre-processing schemes with several images to produce feature vector
	Extreme machine-learning [39]	Machine learning with many discriminative features	Morphology and other conventional approaches are needed to produce discriminative features
	Mahalanobis distance classifier [40]	Simple procedure for training	Pre-processing overhead is still required to compute relevant features
	U-Net-based CNN for semantic segmentation [43]	U-Net structure preserves the boundaries well	Gray scale pre-processing is required
	Multi-scale CNN [44,47]	Better learning due to multi-receptive fields	Tiny vessels not detected in certain cases
	CNN with CRFs [45]	CNN with few layers provides faster segmentation	CRFs are computationally complex
	SegNet-inspired method [46]	Encoder and decoder architecture provides a uniform structure of network layers	Use of PCA to prepare data for training
	CNN with visual codebook [48]	10-layer CNN for correlation with ground truth representation	No end-to-end system for training and testing
	CNN with quantization and pruning [49]	Pruned convolutions increase the efficiency of the network	Fully connected layers increase the number of trainable parameters
	Three-stage CNN-based deep-learning method [50]	Fusion of multi-feature image provides powerful representation	Usage of three CNNs requires more computational power and cost
	Modified U-Net with dice loss [51]	Dice loss provides good results with unbalanced classes	Use of PCA to prepare data for training
	Deformable U-Net-based method [52]	Deformable networks can adequately accommodate geometric variations of data	Patch-based training and testing is time-consuming
	PixelBNN [53]	Pixel CNN is famous for predicting pixels with spatial dimensions	Use of CLAHE for pre-processing
	Dense U-Net-based method [54]	Dense block is good for alleviating vanishing gradient problem	Patch-based training and testing is time-consuming
	Cross-connected CNN (CcNet) [55]	Cross-connections of layers empower features	Complex architecture with pre-processing
	Vess-Net (this work)	Robust segmentation with fewer layers	Augmented data necessary to fully train network

**Table 2:** Key differences between Vess-Net and previous architectures.

Method	Other Architectures	Vess-Net
ResNet [58]	Residual skip path between adjacent layers only	Skip connections between adjacent layers and directly between the encoder and decoder
	All variants use 1 × 1 convolution bottleneck layer	1 × 1 convolution is used only in non-identity residual paths in combination with BN
	No index information with max-pooling layers	Index information between max-pooling and max-unpooling layers used to maintain feature size and location
	One fully connected layer for classification network	Fully convolutional network (FCN) for semantic segmentation, so fully connected layers not used
	Average pooling at the end in place of max-pooling in each block	Max-pooling in each encoder block and max-unpooling in each decoder block
IrisDenseNet [62]	Total of 26 (3 × 3) convolutional layers	Total of 16 (3 × 3) convolutional layers
	Uses dense connectivity inside each dense block via feature concatenation in just encoder	Uses residual connectivity via element-wise addition
	Different number of convolutional layers: first two blocks have two convolutional layers and other blocks have three	Same number of convolutional layers (two layers) in each block of encoder and decoder
	No dense connectivity inside decoder	Uses connectivity inside and between the encoder and decoder
FRED-Net [61]	Only uses residual connectivity between adjacent layers	Uses residual connectivity between adjacent layers in encoder and decoder and residual connectivity outside encoder and decoder
	No outer residual skip paths for direct spatial edge information flow	Inner and outer residual connections for information flow
	Total of 6 skip connections inside the encoder and decoder	10 residual skip connections in encoder and decoder
	Only non-identity mapping for residual connections	Non-identity mapping for inner residual connections (Stream 1) and identity mapping for outer residual connections (Stream 2)
	Only uses post-activation because ReLU activation is used after element-wise addition	Uses the pre-activation and post-activation as ReLU is used before and after the element-wise addition

**Table 3:** Vess-Net encoder with inner and outer residual skip connections and activation size of each layer (ECB, ECon, ORSP, IRSP, and Pool indicate encoder convolutional block, encoder convolution layer, outer residual kip path, internal residual skip path, and pooling layer, respectively). “**” refers to layers that include both batch normalization (BN) and ReLU. “*” refers to layers that include only BN, and *Pool/*Unpool shows that the pooling/unpooling layer is activated prior to the ReLU layer. Outer residual skip paths (ORSP-1 to ORSP-4) are initiated from the encoder to provide spatial information to the decoder. Vess-Net uses both pre- and post-activation. The table is based on the digital retinal images for vessel extraction (DRIVE) dataset, which has a size of 447 × 447 × 3.

Block	Name/Size	Number of Filters	Output Feature Map Size (Width × Height × Number of Channels)	Number of Trainable Parameters (Econ + BN)
**ECB-1**	ECon-1_1 **/3 × 3 × 3 To decoder (ORSP-1)	64	447 × 447 × 64	1792 + 128
	ECon-1_2 **/3 × 3 × 64	64		36,928 + 128
**Pooling-1**	Pool-1/2 × 2	-	223 × 223 × 64	-
**ECB-2**	ECon-2_1 **/3 × 3 × 64 To decoder (ORSP-2)	128	223 × 223 × 128	73,856 + 256
	IRSP-1 */1 × 1 × 64	128		8320 + 256
	ECon-2_2 */3 × 3 × 128	128		147,584 + 256
	Add-1 (ECon-2_2* + IRSP-1 *)	-		-
**Pooling-2**	* Pool-2/2 × 2	-	111 × 111 × 128	-
**ECB-3**	ECon-3_1 **/3 × 3 × 128 To decoder (ORSP-3)	256	111 × 111 × 256	295,168 + 512
	IRSP-2 */1 × 1 × 128	256		33,024 + 512
	ECon-3_2 */3 × 3 × 256	256		590,080 + 512
	Add-2 (ECon-3_2 * + IRSP-2 *)	-		-
**Pooling-3**	* Pool-3/2×2	-	55 × 55 × 256	-
**ECB-4**	ECon-4_1 **/3 × 3 × 256 To decoder (ORSP-4)	512	55 × 55 × 512	1,180,160 + 1024
	IRSP-3 */1 × 1 × 256	512		131,584 + 1024
	ECon-4_2 */3 × 3 × 512	512		2,359,808 + 1024
	Add-3 (ECon-4_2 * + IRSP-3 *)	-		-
**Pooling-4**	* Pool-4/2 × 2	-	27 × 27 × 512	-

**Table 4:** Vess-Net decoder with inner and outer residual skip connections and activation size of each layer (DCB, DCon, ORSP, IRSP, and Unpool indicate decoder convolutional block, decoder convolution layer, outer residual skip path, internal residual skip path, and unpooling layer, respectively). “**” refers to layers that include both BN and ReLU. “*” refers to layers with only BN, and *Pool/*Unpool shows that the pooling/unpooling layer is activated prior to the ReLU layer. Outer residual skip paths (ORSP-1 to ORSP-4) are initiated from the encoder to provide spatial information to the decoder. Vess-Net uses both pre- and post-activation. The table is based on the DRIVE dataset, which has a size of 447 × 447 × 3.

Block	Name/Size	Number of Filters	Output Feature Map Size (Width × Height × Number of Channels)	Number of Trainable Parameters (DCon + BN)
**Un-pooling-4**	Unpool-4	-	55 × 55 × 512	-
**DCB-4**	DCon-4_2 **/3 × 3 × 512	512		2,359,808 + 1024
	ORSP-4 from encoder ECon-4_1 ** Add-4 (DCon-4_2 ** + ECon-4_1 **)	-		-
	IRSP-4 */1 × 1 × 512	256	55 × 55 × 256	131,328 + 512
	DCon-4_1 */3 × 3 × 512	256		1,179,904 + 512
	Add-5 (DCon-4_1 * + IRSP-4 *)	-		-
**Unpooling-3**	* Unpool-3	-	111 × 111 × 256	-
**DCB-3**	DCon-3_2 **/3 × 3 × 256	256		590,080 + 512
	ORSP-3 from encoder ECon-3_1 ** Add-6 (DCon-3_2 ** + ECon-3_1 **)	-		-
	IRSP-5 */1 × 1 × 256	128	111 × 111 × 128	32,896 + 256
	DCon-3_1 **/3 × 3 × 256	128		-
	Add-7 (DCon-3_1 * + IRSP-5 *)	-		-
**Unpooling-2**	* Unpool-2	-	223 × 223 × 128	-
**DCB-2**	DCon-2_2 **/3 × 3 × 128	128		147,584 + 256
	ORSP-2 from encoder ECon-2_1 ** Add-8 (DCon-2_2 ** + ECon-2_1 **)	-		-
	IRSP-6 */1 × 1 × 128	64	223 × 223 × 64	8256 + 128
	DCon-2_1 **/3 × 3 × 128	64		73,792 + 128
	Add-9 (DCon-3_1 * + IRSP-6*)	-		-
**Unpooling-1**	* Unpool-1	-	447 × 447 × 64	-
**DCB-1**	DConv-1_2 **/3 × 3 × 64	64		36,928 + 128
	ORSP-1 from encoder ECon-1_1 ** Add-10(DConv-1_2 **+ ECon-1_1 **)	-		-
	DConv-1_1 **/3 × 3 × 64	2	447 × 447 × 2	1154 + 4

**Table 5:** Accuracies of Vess-Net and existing methods for DRIVE dataset (unit: %).

Type	Method	Se	Sp	AUC	Acc
Handcrafted local feature-based methods	Akram et al. [23]	-	-	96.32	94.69
	Fraz et al. [24]	74.0	98.0	-	94.8
	Kar et al. [25]	75.48	97.92	-	96.16
	Zhao et al. (without Retinex) [26]	76.0	96.8	86.4	94.6
	Zhao et al. (with Retinex) [26]	78.2	97.9	88.6	95.7
	Pandey et al. [27]	81.06	97.61	96.50	96.23
	Neto et al. [28]	78.06	96.29	-	87.18
	Sundaram et al. [29]	69.0	94.0	-	93.0
	Zhao et al. [30]	74.2	98.2	86.2	95.4
	Jiang et al. [31]	83.75	96.94	-	95.97
	Rodrigues et al. [32]	71.65	98.01	-	94.65
	Sazak et al. [33]	71.8	98.1	-	95.9
	Chalakkal et al. [34]	76.53	97.35	-	95.42
	Akyas et al. [36]	74.21	98.03	-	95.92
Learned/deep feature-based methods	Zhang et al. (without post-processing) [37]	78.95	97.01	-	94.63
	Zhang et al. (with post-processing) [37]	78.61	97.12	-	94.66
	Tan et al. [38]	75.37	96.94	-	-
	Zhu et al. [39]	71.40	98.68	-	96.07
	Wang et al. [40]	76.48	98.17	-	95.41
	Tuba et al. [41]	67.49	97.73	-	95.38
	Girard et al. [43]	78.4	98.1	97.2	95.7
	Hu et al. [44]	77.72	97.93	97.59	95.33
	Fu et al. [45]	76.03	-	-	95.23
	Soomro et al. [46]	74.6	91.7	83.1	94.6
	Guo et al. [47]	78.90	98.03	98.02	95.60
	Chudzik et al. [48]	78.81	97.41	96.46	-
	Yan et al. [50]	76.31	98.20	97.50	95.38
	Soomro et al. [51]	73.9	95.6	84.4	94.8
	Jin et al. [52]	79.63	98.00	98.02	95.66
	Leopold et al. [53]	69.63	95.73	82.68	91.06
	Wang et al. [54]	79.86	97.36	97.40	95.11
	Feng et al. [55]	76.25	98.09	96.78	95.28
	Vess-Net (this work)	80.22	98.1	98.2	96.55

**Table 6:** Accuracies of Vess-Net and existing methods for CHASE-DB1 dataset (unit: %).

Type	Method	Se	Sp	AUC	Acc
Handcrafted local feature-based methods	Fraz et al. [24]	72.2	74.1	-	94.6
	Pandey et al. [27]	81.06	95.30	96.33	94.94
	Sundaram et al. [29]	71.0	96.0	-	95.0
Learned/deep feature-based methods	Zhang et al. (without post-processing) [37]	77.86	96.94	-	94.97
	Zhang et al. (with post-processing) [37]	76.44	97.16	-	95.02
	Wang et al. [40]	77.30	97.92	-	96.03
	Fu et al. [45]	71.30	-	-	94.89
	Yan et al. [50]	76.41	98.06	97.76	96.07
	Jin et al. [52]	81.55	97.52	98.04	96.10
	Leopold et al. [53]	86.18	89.61	87.90	89.36
	Vess-Net (this work)	82.06	98.41	98.0	97.26

**Table 7:** Accuracies of Vess-Net and existing methods for STARE dataset (unit: %).

Type	Method	Se	Sp	AUC	Acc
Handcrafted local feature-based methods	Akram et al. [23]	-	-	97.06	95.02
	Fraz et al. [24]	75.54	97.6	-	95.3
	Kar et al. (normal cases) [25]	75.77	97.88	-	97.30
	Kar et al. (abnormal cases) [25]	75.49	96.99	-	97.41
	Zhao et al. (without Retinex) [26]	76.6	97.72	86.9	94.9
	Zhao et al. (with Retinex) [26]	78.9	97.8	88.5	95.6
	Pandey et al. [27]	83.19	96.23	95.47	94.44
	Neto et al. [28]	83.44	94.43	-	88.94
	Zhao et al. [30]	78.0	97.8	87.4	95.6
	Jiang et al. [31]	77.67	97.05	-	95.79
	Sazak et al. [33]	73.0	97.9	-	96.2
Learned/deep feature-based methods	Zhang et al. (without post-processing) [37]	77.24	97.04	-	95.13
	Zhang et al. (with post-processing) [37]	78.82	97.29	-	95.47
	Wang et al. [40]	75.23	98.85	-	96.40
	Hu et al. [44]	75.43	98.14	97.51	96.32
	Fu et al. [45]	74.12	-	-	95.85
	Soomro et al. [46]	74.8	92.2	83.5	94.8
	Chudzik et al. [48]	82.69	98.04	98.37	-
	Hajabdollahi et at. (CNN) [49]	78.23	97.70	-	96.17
	Hajabdollahi et at. (Quantized CNN) [49]	77.92	97.40	-	95.87
	Hajabdollahi et at. (Pruned-quantized CNN) [49]	75.99	97.57	-	95.81
	Yan et al. [50]	77.35	98.57	98.33	96.38
	Soomro et al. [51]	74.8	96.2	85.5	94.7
	Jin et al. [52]	75.95	98.78	98.32	96.41
	Leopold et al. [53]	64.33	94.72	79.52	90.45
	Wang et al. [54]	79.14	97.22	97.04	95.38
	Feng et al. [55]	77.09	98.48	97.0	96.33
	Vess-Net (this work)	85.26	97.91	98.83	96.97

**Table 8:** Accuracies of Vess-Net trained on DRIVE and CHASE-DB1, and tested on STARE dataset (unit: %).

Method	Se	Sp	AUC	Acc
Vess-Net (this work)	81.13	96.21	97.4	95.11
